# Stigma and Associated Correlates of Elderly Patients With Parkinson's Disease

**DOI:** 10.3389/fpsyt.2021.708960

**Published:** 2021-07-15

**Authors:** Miaomiao Hou, Xiaowei Mao, Xiaojun Hou, Kunpeng Li

**Affiliations:** ^1^Department of Neurology, The First Affiliated Hospital of Naval Medical University, Shanghai, China; ^2^Department of Neurorehabilitation, The Second Rehabilitation Hospital of Shanghai, Shanghai, China; ^3^School of Rehabilitation Science, Shanghai University of Traditional Chinese Medicine, Shanghai, China

**Keywords:** Parkinson's disease, stigma, SSCI, depression, predictor

## Abstract

**Background:** Stigmatizing experiences is common in Parkinson's disease (PD) and appears to provide a negative contribution to the quality of life. Our aim of this study was to investigate the extent of stigma and its predictive factors in patients with PD from our hospital in Shanghai, China.

**Methods:** In 276 individuals with PD (135 women and 141 men), stigma was measured by the 24-item Stigma Scale for Chronic Illness (SSCI). Multivariate linear regression model was used to assess predictors of stigma including demographics (age and gender), disease duration, stage (Hoehn and Yahr Scale), motor function (Unified Parkinson's Disease Rating Scale Part 3, UPDRS-III), non-motor symptoms (Non-Motor Symptoms Scale, NMSS), cognitive level (Mini-Mental State Examination, MMSE), as well as anxiety (Hamilton Anxiety Rating Scale, HAM-A) and depressive disorders (Hamilton Depression Rating Scale, HAM-D-24).

**Results:** The total score of SSCI was 49.9 ± 14.3, and 48.5% of the patients checked “rarely” to “sometimes.” For the total sample, the full model accounted for 47.8% of the variance in stigma (*P* < 0.05). Higher UPDRS-III scores, longer course of disease, younger age, tremor-dominant subtype, and higher depression scores were significantly associated with stigma among individuals with PD.

**Conclusion:** Our finding suggested a mild-to-moderate level of stigma in patients with PD. Tremor-dominant subtype, longer course of disease, younger age, severe motor symptoms, and depression are the predictors of stigma in PD.

## Introduction

Parkinson's disease (PD) is the second most common neurodegenerative disease, affecting ~1% of the population over 60 years of age (1). Although PD is traditionally considered to be a motor disorder, the burden of the disease extends far beyond physical impairments. An important issue is the stigma experienced by the individuals with PD (2, 3).

In 1963, Erving Goffman defined stigma as “the situation of the individual who is disqualified from full social acceptance (Preface),” and since then, social scientists have studied the stigma manifested as stereotypes, prejudice, and discrimination (4). The social identity of the stigmatized person may be deeply threatened and damaged (5). The stigma of neuropsychiatric diseases can lead to a variety of adverse consequences, including delays in seeking medical help, diagnosis, and treatment; a low quality of life; failure to adhere to treatment; and increased suicide rates (6–9).

With regard to PD, previous evidence has shown that more than half of the patients with PD are likely to try to conceal their diagnosis (10). Furthermore, there is a high prevalence of stigma in PD patients who attempted to mask their clinical symptoms (11).

There are many reasons and associated factors for the stigma in patients with PD. Stigma may result from motor symptoms such as facial masking, which can increase negative experience in social relationship, even among those trained healthcare providers (12). Meanwhile, invisible stigma is the realization of a self with PD, a form of disability, which attested to the mounting isolation (11). Recently, a few studies revealed that difficulties in activities of daily living, younger age, and higher depression scores were the significant predictors of stigma (13, 14). However, relative studies are limited in China.

Considering the differences between Chinese and Western cultures, the stigma of patients with PD may vary according to different demographic and disease characteristics. The aim of this study was to investigate the extent of stigma and its associated factors in Chinese patients with PD on their early to middle stages of the disease.

## Materials and Methods

### Subjects

The protocol for this study was reviewed and approved by the ethics committee of the First Affiliated Hospital of Naval Medical University. Informed consent was obtained from all participants prior to participation in this study. Patients were recruited from the First Affiliated Hospital of Naval Medical University. Inclusion criteria were as follows: (1) the diagnosis of idiopathic Parkinson's disease according to the UK Brain Bank Criteria; (2) age ≥50 years old; and (3) a modified Hoehn and Yahr stage less than or equal to 4. The exclusion criteria included a diagnosis of other serious physical defects, cognitive impairment, or unable to complete the investigation. A total of 276 patients with idiopathic Parkinson's disease treated in our hospital were enrolled.

### Procedures and Measures

Patients were interviewed face to face by neurologists. The Unified Parkinson's Disease Rating Scale (UPDRS III) was applied to assess PD motor symptoms. The disease stage was evaluated by using the Hoehn and Yahr Scale. Motor symptom subtype was calculated by the ratio of mean tremor to mean postural instability and gait difficulty (PIGD) symptoms. For the original UPDRS, a ratio ≥1.5 was classified as tremor dominant (TD), a ratio ≤1.0 was classified as PIGD, and a ratio between 1.0 and 1.5 was classified as indeterminate (IND) (15, 16).

Anxiety and depression symptoms were assessed by using the Hamilton Anxiety Rating Scale (HAM-A) and Hamilton Depression Rating Scale (HAM-D-24), respectively. The cognitive level was evaluated by the Mini-Mental State Examination (MMSE). The severity of non-motor symptoms was assessed by the Non-Motor Symptoms Scale (NMSS).

The 24-item Stigma Scale for Chronic Illness (SSCI) was developed to measure stigma experienced by individuals with chronic neurological disorders including PD. It contains two subscales: felt stigma and enacted stigma (17). The felt stigma subscale (13 items) asks questions about the respondent's feelings (e.g., embarrassment, worry, and self-blame). The enacted stigma subscale (11 items) asks questions about the behavior of others toward the respondent (e.g., avoiding contact, staring, and being unkind). Each item is rated as 1 = never, 2 = rarely, 3 = sometimes, 4 = often, and 5 = always. A higher score indicates a higher frequency of experiencing stigma. A systematic review suggests that the SSCI has good content validity and enough internal consistency (18). All the assessors were trained before the start of the study. Inter-rater concordance of all assessments was more than 0.8.

### Statistical Analysis

The Kolmogorov–Smirnov test was applied to detect the distribution normality of variables. Residual plot was applied for equivalence of variance. Continuous variables were represented as mean and standard deviation. Categorical variables were described as absolute numbers, median, and frequencies. Pearson and Spearman correlations were conducted for continuous variable and categorical ones, respectively, to investigate the association of demographic and clinical characteristics with SSCI score.

Significant correlates were entered as predictors in multivariate linear regression model with SSCI as the dependent variable. The α level of significance was set to *p* < 0.05 (two-tailed). All analyses were performed by IBM SPSS statistics 20 for Windows.

## Results

As shown in Table 1, the patients recruited in this study had an average age of 62.5 years (*SD* = 8.5) and disease duration of 9.9 years (*SD* = 4.9). Median-modified Hoehn and Yahr (H&Y) stage was 3 (range 1–4). The average motor severity as measured by the UPDRS III was 23.9 (*SD* = 11.2). Motor symptom subtypes consisted of 92 with TD profile (33.3%), 121 characterized by PIGD (43.8%), and 63 IND (22.8%), all as per time of assessment. Each participant had a MMSE score more than 24. Up to 80 patients (28.9%) had HAM-D-24 score above 20, suggesting mild to moderate depression. The mean SSCI score was 49.9 (*SD* = 14.3, range: 24–96). The scores of felt stigma and enacted stigma were 29.4 (*SD* = 9.5) and 20.6 (*SD* = 9.7), respectively. For the whole group, the significant correlates of SSCI were age, disease duration, MMSE, NMSS, HAM-D-24, HAM-A, UPDRS III, TD, and PIGD (*P* < 0.05).

**Table 1:** General characteristics of PD patients.

**Variables**	**Mean ± SD**
Gender *n* (%)	
Female	135 (48.7)
Male	141 (50.9)
Age	62.5 ± 8.5
Education (years)	10.8 ± 4.1
Disease duration (years)	9.9 ± 4.9
UPDRS-III	23.9 ± 11.3
Motor symptom subtype, *n* (%)	
TD	92 (33.3)
PIGD	121 (43.8)
IND	63 (22.8)
MMSE	26.7 ± 3.1
NMSS	17.6 ± 5.3
HAM-D-24	16.7 ± 6.4
HAM-A	14.7 ± 3.8
H-Y, *n* (%)	
I	15 (5.4)
II	78 (28.2)
III	140 (50.6)
VI	43 (15.5)
Levodopa equivalent dose (mg/day)	868.6 ± 485.0
SSCI	2.1 ± 1.1
Below rarely (*n*, %)	142, 51.4%
Rarely to below sometimes (*n*, %)	119, 43.1%
Sometimes to below often (*n*, %)	15, 5.4%
Felt stigma	2.3 ± 1.0
Below rarely (*n*, %)	114, 41.3%
Rarely to below sometimes (*n*, %)	125, 45.3%
Sometimes to below often (*n*, %)	34, 12.3%
Often to below always (*n*, %)	5, 1.8%
Enacted stigma	1.9 ± 1.0
Below rarely (*n*, %)	188, 68.1%
Rarely to below sometimes (*n*, %)	58, 21.0%
Sometimes to below often (*n*, %)	29, 10.5%

*SCCI, Stigma Scale for Chronic Illness; H-HY; Hoehn and Yahr Scale; UPDRS-III, Unified Parkinson's Disease Rating Scale Part 3; NMSS, Non-Motor Symptoms Scale; MMSE, Mini-Mental State Examination; HAM-A, Hamilton Anxiety Rating Scale; HAM-D-24, Hamilton Depression Rating Scale; PIGD, postural instability and gait difficulty; TD, tremor dominant; IND, indeterminate*.

Multivariate linear regression model (Table 2) showed that SSCI score was significantly associated with UPDRS III, TD, HAM-D-24, disease duration, and age. The full model accounted for 47.8% of the variance in SSCI score (*P* < 0.05). We conducted multicollinearity analysis in the regression model to ensure that the contribution of each aspect was independent. The variance inflation factor was below 2 and the condition index was lower than 30. Felt stigma was significantly associated with UPDRS III, HAM-D-24, and disease duration. This model accounted for 67.3% of the variance. For enacted stigma, the significant correlates were UPDRS III, TD, and age, which together accounted for 23.2% of the variance.

**Table 2:** Multivariate linear regression model of factors associated with SSCI.

**Variables**	***R***	**Adjusted *R*^**2**^**	***F***	**Coefficient β (95% CI)**	***P-*value**
SSCI	0.699	0.478	46.04		
UPDRS-III				0.79 (0.66~0.91)	<0.001
TD				6.61 (3.76~9.46)	<0.001
HAM-D-24				0.49 (0.24~0.74)	<0.001
Disease duration				0.42 (0.003~1.41)	0.003
Age				−0.17 (0.04~-0.33)	0.043
Felt stigma	0.674	0.447	67.31		
UPDRS-III				0.47 (0.40~0.55)	<0.001
HAM-D-24				0.39 (0.24~0.55)	0.001
Disease duration				0.31 (0.13~0.49)	0.004
Enacted stigma	0.526	0.277	23.18		
UPDRS-III				0.34 (0.25~0.43)	0.001
TD				5.02 (3.03~7.01)	0.003
Age				−0.18 (−0.29~-0.06)	0.035

*SCCI, Stigma Scale for Chronic Illness; UPDRS-III, Unified Parkinson's Disease Rating Scale Part 3; HAM-D-24, Hamilton Depression Rating Scale; TD, tremor dominant*.

Multivariate linear regression model was also conducted for felt and enacted SSCI according to gender (Table 3). Felt stigma was associated in female with higher UPDRS III and HAM-D-24 and in male with higher UPDRS III, longer disease duration, and HAM-D-24. For enacted stigma, the significant correlates in female were higher UPDRS III and younger age, while in male, it included TD and UPDRS III.

**Table 3:** Multivariate linear regression model of factors associated with felt and enacted SSCI according to gender.

**Variables**	***R***	**Adjusted *R*^**2**^**	***F***	**Coefficient β (95% CI)**	***P*-value**
Felt stigma					
In female	0.565	0.308	28.77		
UPDRS-III				0.47 (0.33~0.61)	<0.001
HAM-D-24				0.28 (0.04~0.53)	0.026
In male	0.780	0.598	60.43		
UPDRS-III				0.48 (0.39~0.57)	<0.001
Disease duration				0.63 (0.39~0.87)	<0.001
HAM-D-24				0.55 (0.35~0.74)	<0.001
Enacted stigma					
In female	0.345	0.105	8.34		
UPDRS-III				0.29 (0.13~0.45)	<0.001
Age				−0.26 (−0.46~-0.07)	0.009
In male	0.704	0.487	57.88		
TD				9.12 (6.68~11.56)	<0.001
UPDRS-III				0.31 (0.22~0.40)	<0.001

*UPDRS-III, Unified Parkinson's Disease Rating Scale Part 3; HAM-D-24, Hamilton Depression Rating Scale; TD, tremor dominant*.

## Discussion

The main findings of our study were that stigma was at a mild-to-moderate level in patients with PD. Tremor-dominant subtype, longer course of disease, younger age, severe motor symptoms, and depression were the significant correlates of stigma in PD. In addition, female and male PD patients had their own factors. These results suggested that we neurologists could better help elderly PD patients cope with stigma by means of improving their motor symptoms, relieving tremor symptoms, and at the same time paying attention to emotional disorders.

Our study found that the UPDRS III was significantly associated with the score of SSCI. The obvious motor symptoms of Parkinson's disease patients, such as static tremor, bradykinesia, and abnormal posture and gait, could come to light in public places. These changes in body image would lead to patients' sense of shame, embarrassment, and isolation (19, 20). One study suggested that the stigma of Parkinson's disease patients was related to the change of their external image and the gradual loss of their functions (20). At the same time, the language communication barrier and the non-verbal communication barrier caused by “facial masking” also inevitably led to the isolation of the patients (11). Patients may be mistaken for concealing their illness and unwilling to communicate with others, which aggravated the sense of isolation (21). Motor symptoms can directly lead to social isolation and self-discrimination, so motor symptoms can be the main source of stigma in patients with Parkinson's disease.

By separating overall SSCI into felt and enacted stigma, we found that felt stigma, compared to enacted stigma, was experienced to a stronger degree (Table 1). Moreover, depression was strongly related to the score of felt SSCI. This finding was in accord with the work by Salazar et al. who recognized that depression was a significant predictor in stigma and depression mediated the relation between stigma and activities of daily living (13). A British study shows that 46% of PD patients stopped working after 5 years of illness, and more than half of them chose to retire early, resulting in economic burden and psychological pressure of patients (22). The change of social role and social interaction exerted a negative impact on the family's quality of life and psychology. As stigma is inseparable from the socio-cultural environment, it is important to recognize the social meaning of PD and PD-related symptoms. The association between depression and stigma perception has important implications for conceptualizing stigma in PD and its potential treatment targets.

Inconsistent with other foreign studies, TD subtype, relative to PIGD or IND, was more likely to have higher enacted SSCI score. Hermanns suggested that facial masking was commonly reported by all the PD participants. This can result in isolation of the stigmatized person (11). However, socio-cultural norms about facial expressivity vary according to culture; a study conducted by Tickle-Degnen et al. found that American practitioners' judgments of patient sociability were more negatively biased in response to facial masking than those of Taiwanese practitioners (12). Unfortunately, our study did not separately list “facial mask” as a predictive factor. While communicating with the participants, we were aware of the frequent mention of “tremor,” especially in male patients, who still demanded to increase the dosage of drugs when their clinical symptoms have been well-controlled. This kind of care about “other people's attitude” has led to self-cognition bias. Caap-Ahlgren and Lannerheim found that female PD patients felt particularly conspicuous because of involuntary movement of their limbs in social intercourse, and they were mistaken for drunkenness. This misunderstanding further strengthens the public's belittling and discriminatory attitude (23).

Younger age was related to increased perception of stigma in PD. The experience of diagnosis of a neurodegenerative disease relatively early in life may be qualitatively different than diagnosis in later life, having a greater impact on self-perception and self-expectations in family, social, and occupational roles (24, 25). Meanwhile, the unpredictability and inability to prevent or slow down the progression of Parkinson's disease may bring uncertainty and psychological pressure to patients (26). With the prolongation of the course of disease, the patients were forced to give up their daily work and social life, aggravating social isolation.

There were some limitations in this study. First, this study is not sufficient to infer the causal relationships between independent variables and outcomes. Secondly, this study lacked the assessment of life ability such as PDQ-39 or ADL, which is widely used in other studies. Also, the social status such as household income, marital status, occupation, or type of the medical insurance were not documented. These may also be the influencing factors in stigma. The last but not the least, the sample was only limited to one hospital and did not consist of severely affected patients; therefore, the promotion of the results has certain limitations.

## Conclusion

This study investigated the stigma in PD patients of different motor subtypes and different stages in our hospital. The results showed that the stigma in PD patients was in the mild-to-moderate level, and the correlated factors were complex, mainly related to the disease progression and emotional disorders. Future research would increase the number of patients from different hospitals and different regions to explore more predictors so that adaptive interventions could be formulated to effectively reduce the stigma and improve the quality of life of PD patients.

## Data Availability Statement

The raw data supporting the conclusions of this article will be made available by the authors, without undue reservation.

## Ethics Statement

The studies involving human participants were reviewed and approved by the Ethics committee of the First Affiliated Hospital of Naval Medical University. The patients/participants provided their written informed consent to participate in this study.

## Author Contributions

KL designed the research. XM and XH examined the patients and collected the data. MH performed the statistical analysis and drafted the manuscript. All authors made contributions to this study, critically reviewed the content, and approved the final version of this article.

## Conflict of Interest

The authors declare that the research was conducted in the absence of any commercial or financial relationships that could be construed as a potential conflict of interest.
