# Supplementary material for: Stigma and Associated Correlates of Elderly Patients With Parkinson's Disease
Source: Front Psychiatry. 2021 Jul 15;12:708960. doi: 10.3389/fpsyt.2021.708960 (PMC8319540; doi:10.3389/fpsyt.2021.708960)
Supplement: Supplementary file 1 [file Data_Sheet_1.PDF]

1.residual plot for equivalence of variance.

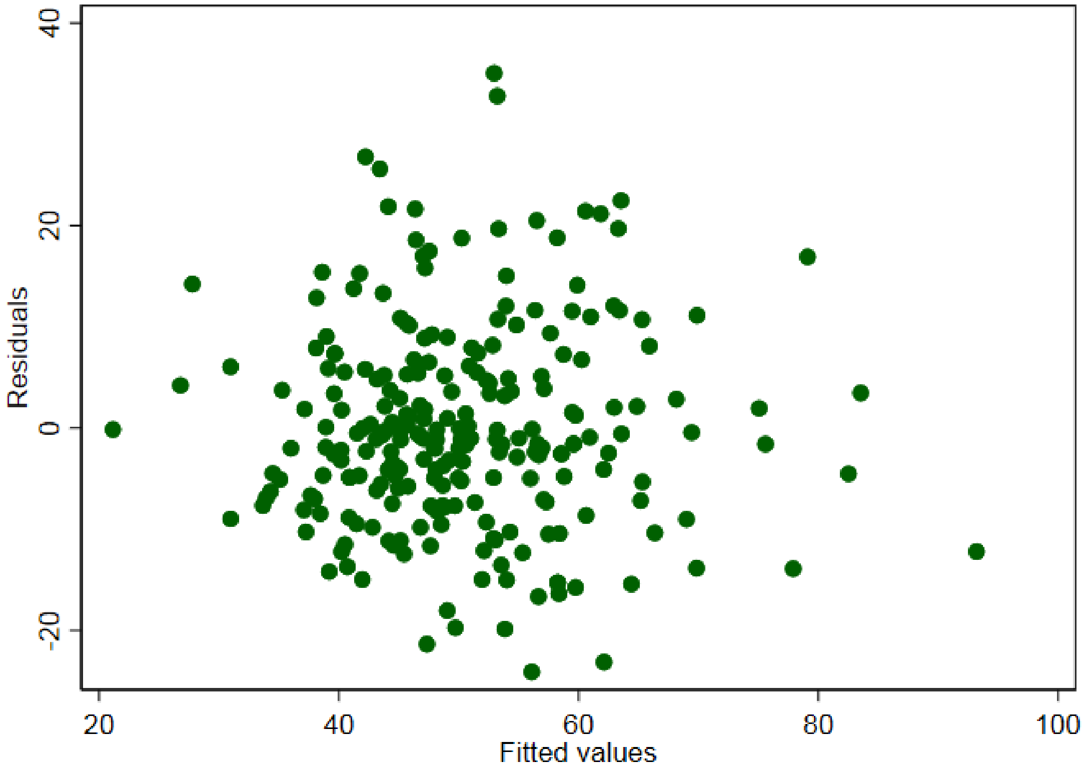

3.Because most variables are continuous ones, we tried PCA. KMO=0.659. From the Correlation Matrix, the information overlap is not significant among the 12 dimensions. From the table Total Variance Explained and the scree plot, the 4 extracted components can not fully interpret the results.

Correlation Matrix

		gender	age	Disease duration	MMSE	NMS	HAMA	HAMD	H-Y	LED	UPDRS III	Motor symptom subtype	SSCI
Correlation	gender	1.000	-.089	-.018	.119	-.116	-.168	-.073	-.117	-.024	.136	-.023	.002
	age	-.089	1.000	.134	-.130	.237	.074	-.080	.093	-.022	.191	.075	.000
	disease duration	-.018	.134	1.000	-.124	.180	.029	-.031	.227	.086	.124	.058	.183
	MMSE	.119	-.130	-.124	1.000	-.226	-.199	-.160	-.218	-.137	-.220	-.129	-.205
	NMS	-.116	.237	.180	-.226	1.000	.492	.463	.276	.216	.094	.207	.127
	HAMA	-.168	.074	.029	-.199	.492	1.000	.753	.385	.200	.094	.054	.213
	HAMD	-.073	-.080	-.031	-.160	.463	.753	1.000	.377	.223	.106	.119	.215
	H-Y	-.117	.093	.227	-.218	.276	.385	.377	1.000	.223	.275	.126	.488
	LED	-.024	-.022	.086	-.137	.216	.200	.223	.223	1.000	-.076	.100	.007
	UPDRS III	.136	.191	.124	-.220	.094	.094	.106	.275	-.076	1.000	.027	.628
	motor symptom subtype	-.023	.075	.058	-.129	.207	.054	.119	.126	.100	.027	1.000	-.089
	SSCI	.002	.000	.183	-.205	.127	.213	.215	.488	.007	.628	-.089	1.000

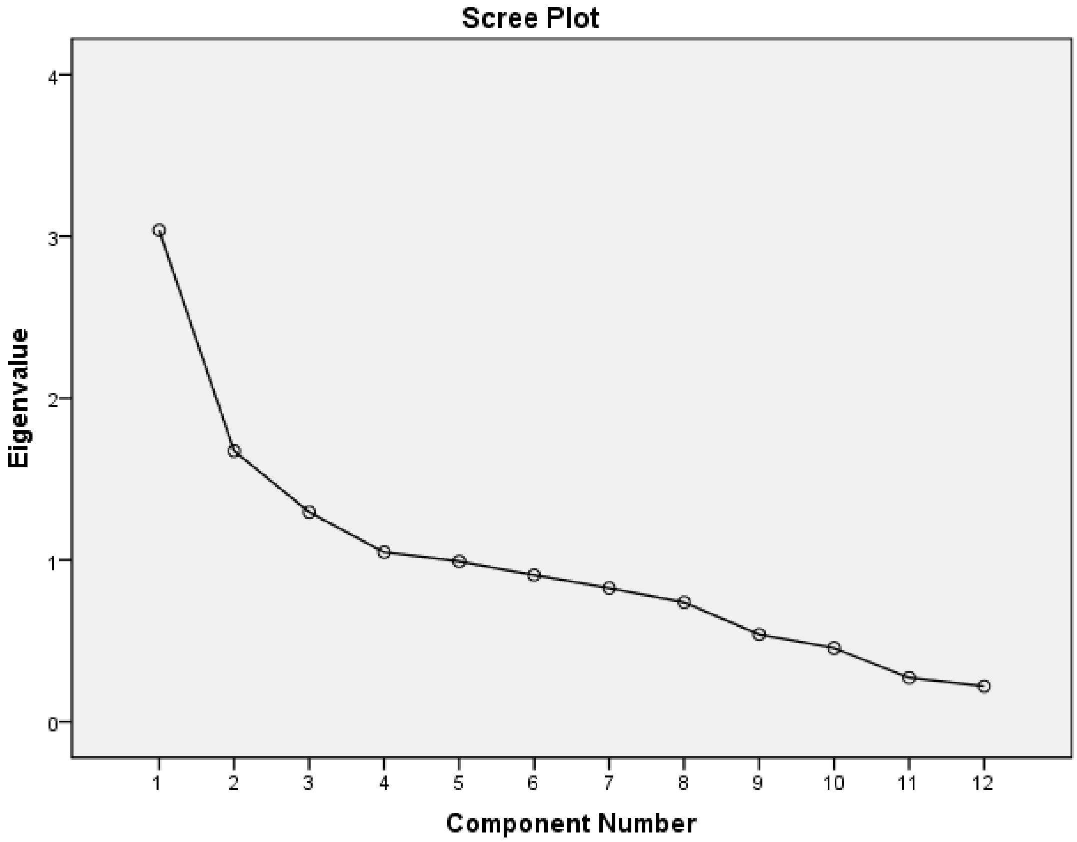

Total Variance Explained

component	Initial Eigenvalues			Extraction sums of squared loadings			Rotation sums of squared loading		
	total	% of variance	Cumulative%	total	% of variance	Cumulative%	total	% of variance	Cumulative%
1	3.038	25.319	25.319	3.038	25.319	25.319	2.395	19.955	19.955
2	1.674	13.948	39.267	1.674	13.948	39.267	2.046	17.051	37.006
3	1.295	10.795	50.062	1.295	10.795	50.062	1.346	11.218	48.224
4	1.048	8.730	58.792	1.048	8.730	58.792	1.268	10.569	58.792
5	.991	8.258	67.050						
6	.906	7.547	74.598						
7	.826	6.883	81.480						
8	.738	6.146	87.626						
9	.539	4.494	92.120						
10	.455	3.789	95.909						
11	.272	2.266	98.175						
12	.219	1.825	100.000						

Extraction method : Principal Component Analysis
